# Monocyte to HDL cholesterol ratio as a marker of the presence and severity of obstructive sleep apnea in hypertensive patients

**DOI:** 10.1038/s41598-021-95095-3

**Published:** 2021-08-04

**Authors:** Min Sun, Chao Liang, Hui Lin, Yuezhi Meng, Qunzhong Tang, Xiaoyu Shi, Erming Zhang, Qiang Tang

**Affiliations:** 1 Department of Cardiology, Peking University Shougang Hospital, Beijing, China; 2 Department of Respiratory, Peking University Shougang Hospital, Beijing, China

**Keywords:** Predictive markers, Hypertension, Sleep disorders

## Abstract

This study aimed to investigate the correlation between monocyte to high-density lipoprotein cholesterol ratio (MHR) and obstructive sleep apnea (OSA) in patients with hypertension. A total of 246 hypertensive patients (67 controls, 65 mild, 51 moderate, and 63 severe OSA) were included. The relationship between MHR and OSA was analyzed. MHR correlated positively with apnea–hypopnea index (AHI), while negatively with mean SpO_2_ (P < 0.01). MHR was higher in OSA group than the control group (9.2 ± 2.6 vs. 10.8 ± 3.6, P < 0.001). Moreover, MHR in severe OSA group was the highest among all groups (9.2 ± 2.6, 10.2 ± 3.2, 10.4 ± 4.0, and 11.8 ± 3.4 in control, mild, moderate, and severe OSA group, respectively, P < 0.001). Logistic regression analysis demonstrated that MHR was an independent predictor of the presence of OSA (OR = 1.152, P < 0.01) and severe OSA (OR = 1.142, P < 0.01). Area under the curve of MHR was 0.634 (P < 0.05) and 0.660 (P < 0.05) for predicting OSA and severe OSA respectively in the ROC analysis. In conclusion, MHR increased with the severity of OSA. As a practical and cost-effective test, MHR was expected to be an available marker in evaluating OSA risk and severity in hypertensive patients.

## Introduction

Obstructive sleep apnea (OSA) is a highly prevalent clinical syndrome affecting more than 10% of the general population^1,2^. It is characterized by recurrent partial or total obstructions of the upper airway during sleep, leading to intermittent hypoxemia (IH) and sleep fragmentation^3^. Accumulating studies reveal that OSA is an independent risk factor for hypertension and consequent cardiovascular morbidities. Additionally, treatment of OSA could greatly improve both OSA symptoms and blood pressure (BP) control. Besides, the therapy was more effective in patients with severe OSA^4^. Therefore, it is of great meaning to detect practical clinical parameters screening the presence and evaluating the severity of OSA in hypertensive patients for early management of OSA, better control of hypertension and further reducing the consequent cardiovascular morbidities.

Increased sympathetic activation, oxidative stress, systemic inflammation, and endothelial dysfunction induced by chronic intermittent hypoxemia (CIH) are taken for the potential mechanism of OSA in leading to the development of hypertension^5,6^. Recently, the monocyte to high density-lipoprotein (HDL) cholesterol ratio (MHR), a new indicator of inflammation and oxidative stress, has been addressed as a predictor and prognostic marker of cardiovascular diseases^7^. Several studies have demonstrated the relationship between MHR and OSA in general population. MHR was found increased with OSA severity^8^, and independently associated with the occurrence of cardiovascular disease in OSA patients as well^9,10^. However, none of the previous studies have ever investigated the association between MHR and OSA in patients with hypertension. Therefore, the aim of the study was to evaluate the association between MHR and OSA, and to further investigate whether MHR could be used as an independent marker to predict OSA presence and severity in hypertensive patients.

## Results

### Demographic characteristics

Initially, 318 participants were enrolled, but 72 participants were excluded from the study because 9 participants were lack of monocyte results or HDL results, 6 participants were lack of complete out of center sleep test (OCST) recordings and 57 participants met the exclusion criteria, such as infection, insomnia, congestive heart failure, chronic obstructive pulmonary disease, etc. Finally, a total of 246 patients were included in the study (183 males, aged 56.7 ± 12.7 years, averaged BMI 27.90 ± 4.43 kg/m^2^), including 179 patients with OSA (OSA group) and 67 patients without OSA (control group).

The baseline characteristics were presented in Tables 1 and 2. The apnea–hypopnea index (AHI), mean oxygen saturation (mean SpO_2_), lowest pulse oxygen saturation (LSpO_2_), the percentage of sleep duration with SpO_2_ < 90% (TS90) and oxygen desaturation index (ODI) were significantly different among all groups (P < 0.05).

**Table 1:** Baseline clinical, laboratory, and OCST data of the study population.

Characteristics	Control (n = 67)	OSA (n = 179)	P value
**Clinical parameters**			
Age (years)	56.6 ± 13.5	56.7 ± 12.4	0.958
Male gender, n (%)	44 (65.7)	139 (77.7)	0.055
Systolic BP (mmHg)	135.6 ± 19.7	136.2 ± 16.0	0.824
Diastolic BP (mmHg)	79.9 ± 13.1	82.6 ± 13.2	0.163
BMI (kg/m^2^)	26.6 ± 3.5	28.4 ± 4.6	0.002*
Obesity, n (%)	43 (64.2)	134 (74.9)	0.068
Alcohol consumption, n (%)	15 (22.4)	44 (24.6)	0.720
Cigarette smoking, n (%)	29 (43.3)	79 (44.1)	0.905
Diabetes mellitus, n (%)	25 (37.3)	54 (30.2)	0.285
Dyslipidemia, n (%)	53 (79.1)	150 (83.8)	0.388
CAD, n (%)	28 (41.8)	104 (58.1)	0.022*
**Medical therapy**			
CCBs, n (%)	33 (49.3)	97 (54.2)	0.490
α- or β-Blockers, n (%)	29 (43.3)	97 (54.2)	0.128
ACEIs/ARBs, n (%)	36 (53.7)	114 (63.7)	0.154
Diuretics, n (%)	9 (13.4)	47 (26.3)	0.033
≥ 3 classes of anti-hypertensive medications, n (%)	14 (20.9)	60 (33.5)	0.055
**Hypertension severity**			
Stage-2 hypertension, n (%)	21 (31.3)	73 (40.8)	0.175
**Laboratory parameters**			
Monocyte count (10^9^/L)	0.4 (0.3–0.4)	0.4 (0.3–0.5)	0.069
Triglyceride (mg/dL)	132.0 (95.2–180.7)	152.4 (111.6–219.7)	0.098
Total cholesterol (mg/dL)	177.8 ± 41.9	173.8 ± 42.3	0.507
HDL cholesterol (mg/dL)	41.7 ± 8.5	38.7 ± 8.2	0.010*
LDL cholesterol (mg/dL)	99.2 ± 30.9	98.3 ± 27.3	0.825
MHR	9.2 ± 2.6	10.8 ± 3.6	< 0.001*
**OCST parameters**			
AHI (events/h)	2.8 ± 1.4	24.8 ± 16.1	< 0.001*
Mean SpO_2_ (%)	94 (93–95)	94 (92–95)	0.018*
LSpO_2_ (%)	82 (80–86)	80 (76–83)	< 0.001*
TS90 (%)	4 (2–38)	23 (7–66)	0.002*
ODI	4.0 ± 4.8	20.5 ± 15.3	< 0.001*

Data are means ± standard deviation, numbers of subjects (%), or medians (range).

*OSA* obstructive sleep apnea, *BP* blood pressure, *BMI* body mass index, *CAD* coronary artery disease, *HDL* high-density lipoprotein, *LDL* low-density lipoprotein, *MHR* monocyte to high-density lipoprotein cholesterol ratio, *OCST* out of center sleep testing, *AHI* apnea–hypopnea index, *Mean* *SpO*_*2*_ mean oxygen saturation, *LSpO*_*2*_ lowest pulse oxygen saturation, *TS90* the percentage of sleep duration with SpO_2_ < 90%, *ODI* oxygen desaturation index.

*P < 0.05.

**Table 2:** Baseline clinical, laboratory, and OCST data of the study population.

Characteristics	Control (n = 67)	Mild OSA (n = 65)	Moderate OSA (n = 51)	Severe OSA (n = 63)	P value
**Clinical parameters**					
Age (years)	56.6 ± 13.5	57.6 ± 11.1	58.3 ± 10.8	54.6 ± 14.6	0.407
Male gender, n (%)	44 (65.7)	48 (73.8)	37 (72.5)	54 (85.7)	0.071
Systolic BP (mmHg)	135.6 ± 19.7	133.4 ± 15.4	138.3 ± 16.8	137.5 ± 15.9	0.410
Diastolic BP (mmHg)	79.9 ± 13.1	80.7 ± 12.0	81.6 ± 12.7	85.3 ± 14.5	0.104
BMI (kg/m^2^)	26.6 ± 3.5	27.5 ± 3.8	28.0 ± 5.0	29.5 ± 4.9*^†^	0.002^#^
Obesity, n (%)	43 (64.2)	47 (72.3)	35 (68.6)	52 (82.5)	0.122
Alcohol consumption, n (%)	15 (22.4)	10 (15.4)	17 (33.3)	17 (27)	0.139
Cigarette smoking, n (%)	29 (43.3)	26 (40.0)	21 (41.2)	32 (50.8)	0.619
Diabetes mellitus, n (%)	25 (37.3)	24 (36.9)	13 (25.5)	17 (27.0)	0.347
Dyslipidemia, n (%)	53 (79.1)	55 (84.6)	44 (86.3)	51 (81.0)	0.718
CAD, n (%)	28 (41.8)	34 (52.3)	32 (62.7)	38 (60.3)	0.084
**Medical therapy**					
CCBs, n (%)	33 (49.3)	35 (53.8)	25 (49.0)	37 (58.7)	0.671
α- or β-Blockers, n (%)	29 (43.3)	30 (46.2)	29 (56.9)	38 (60.3)	0.165
ACEIs/ARBs, n (%)	36 (53.7)	41 (63.1)	31 (60.8)	42 (66.7)	0.483
Diuretics, n (%)	9 (13.4)	14 (21.5)	10 (19.6)	23 (36.5)	0.015^#^
≥ 3 classes of anti-hypertensive medications, n (%)	14 (20.9)	19 (29.2)	13 (25.5)	28 (44.4)	0.024^#^
**Hypertension severity**					
Stage-2 hypertension, n (%)	21 (31.3)	23 (35.4)	16 (31.4)	34 (54.0)	0.027^#^
**Laboratory parameters**					
Monocyte count (10^9^/L)	0.4 (0.3–0.4)	0.4 (0.3–0.4)	0.4 (0.3–0.5)	0.4 (0.3–0.5)*	0.022^#^
Triglyceride (mg/dL)	132.0 (95.2–180.7)	147.1 (106.3–201.1)	168.3 (120.1–252.5)	151.5 (110.3–237.9)	0.262
Total cholesterol (mg/dL)	177.8 ± 41.9	169.3 ± 38.2	179.2 ± 41.4	174.2 ± 46.9	0.566
HDL cholesterol (mg/dL)	41.7 ± 8.5	37.8 ± 7.3*	41.6 ± 9.3	37.2 ± 7.6*	0.001^#^
LDL cholesterol (mg/dL)	99.2 ± 30.9	95.3 ± 25.0	100.2 ± 27.3	99.9 ± 29.7	0.754
MHR	9.2 ± 2.6	10.2 ± 3.2	10.4 ± 4.0	11.8 ± 3.4*^†§^	< 0.001^#^
**OCST parameters**					
AHI (events/h)	2.8 ± 1.4	9.6 ± 4.4*	22.7 ± 4.6*^†^	42.3 ± 12.4*^†§^	< 0.001^#^
Mean SpO_2_ (%)	94 (93–95)	94 (93–95)	94 (93–95)	93 (92–95)*^§^	0.002^#^
LSpO_2_ (%)	82 (80–86)	81 (78–83)	80 (76–83)	77 (68–82)*^†^	< 0.001^#^
TS90 (%)	4 (2–38)	12 (4–30)	19 (7–44)	55 (18–133)*^†§^	< 0.001^#^
ODI	4.0 ± 4.8	8.6 ± 4.4*	17.2 ± 7.9*^†^	35.4 ± 14.9*^†§^	< 0.001^#^

Data are means ± standard deviation, numbers of subjects (%), or medians (range).

*OSA* obstructive sleep apnea, *BP* blood pressure, *BMI* body mass index, *CAD* coronary artery disease, *HDL* high-density lipoprotein, *LDL* low-density lipoprotein, *MHR* monocyte to high-density lipoprotein cholesterol ratio, *OCST* out of center sleep testing, *AHI* apnea–hypopnea index, *Mean* *SpO*_*2*_, mean oxygen saturation, *LSpO*_*2*_ lowest pulse oxygen saturation, *TS90* the percentage of sleep duration with SpO_2_ < 90%, *ODI* oxygen desaturation index.

*vs. Control, P < 0.05; ^†^vs. mild OSA, P < 0.05; ^§^vs. moderate OSA, P < 0.05; ^#^P < 0.05.

**Figure 1:**
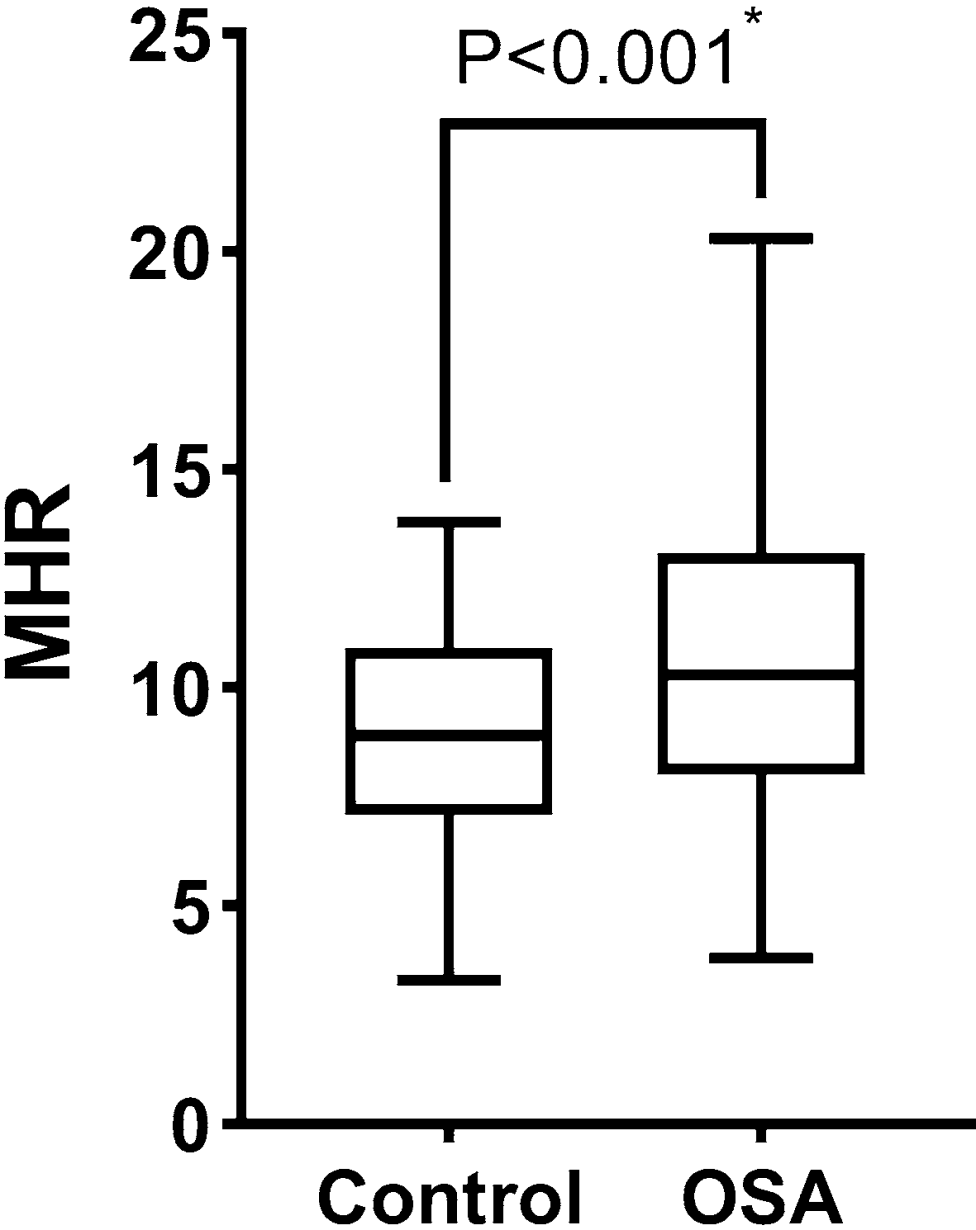
MHR levels in control group and the OSA group. *MHR* monocyte to high-density lipoprotein cholesterol ratio, *OSA* obstructive sleep apnea. *P value < 0.05.

Compared with the control group, body mass index (BMI) value and the prevalence of coronary artery disease (CAD) were higher (P = 0.002 and P = 0.022, respectively) while the level of HDL cholesterol was lower in the OSA group (P < 0.05; Table 1). The mean MHR value was significantly higher in the OSA group (P < 0.001; Fig. 1).

**Figure 2:**
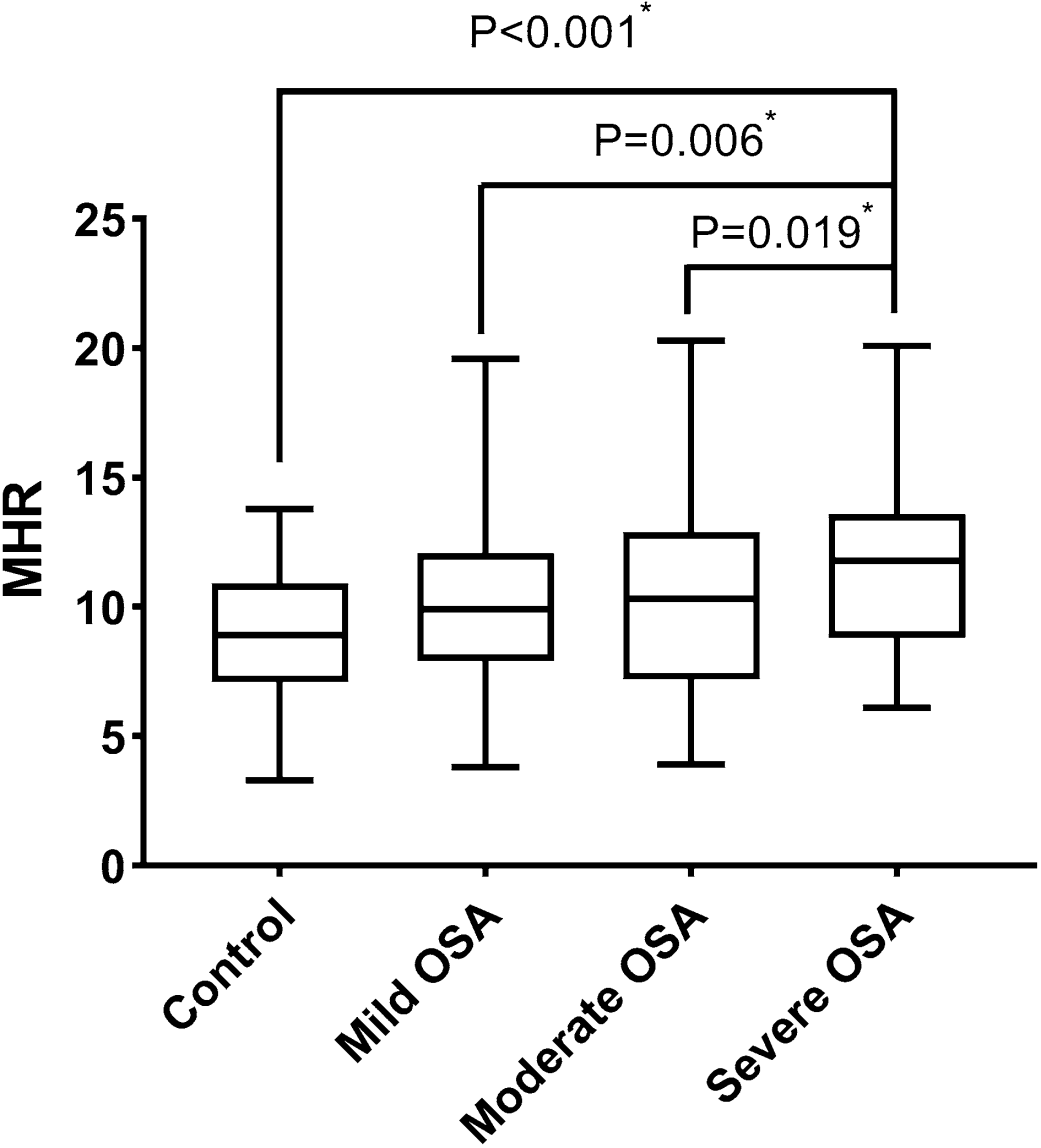
MHR levels in control group, the mild OSA group, the moderate group and the severe group. *MHR* monocyte to high-density lipoprotein cholesterol ratio, *OSA* obstructive sleep apnea. *P value < 0.05.

The OSA group was further categorized into the mild (AHI: 5–14.9), moderate (AHI: 15–29.9), and severe (AHI ≥ 30) OSA group. The mean AHI value of OSA group was 24.8/h, and the prevalence of mild (n = 65), moderate (n = 51), and severe (n = 63) OSA were 26.4%, 20.7%, and 25.6%, respectively. As shown in Fig. 2, MHR was found elevated in parallel with the increase of OSA severity. The level of MHR was significantly higher in the severe OSA group than those in the control group (P < 0.001), the mild OSA group (P = 0.006), and the moderate OSA group (P = 0.019). The BMI value was higher in the severe OSA group when compared with the control group (P < 0.001) and the mild OSA group (P = 0.008; Table 2). Monocyte count was higher in the severe OSA group than the control group (P = 0.048; Table 2). Serum HDL cholesterol level in severe OSA group was the lowest among all 4 groups (P = 0.026; Table 2). In addition, the systolic BP and diastolic BP increased with the severity of OSA although there was no statistical significance which might be contributed to the intensive anti-hypertensive medication management in OSA group (Table 2). Patients in the severe OSA group achieved the highest percentage of ≥ 3 classes of anti-hypertensive medications among all 4 groups (Table 2). Taken both the level of BP and the use of anti-hypertensive medications into consideration of hypertension severity, the percentage of stage-2 hypertension was the highest in the severe OSA group (54.0%, P = 0.027, Table 2), indicating that hypertension severity increased with OSA severity.

### The association between MHR and OSA

As shown in Table 3, MHR was positively correlated with AHI (r = 0.244, P < 0.001), ODI (r = 0.250, P < 0.001), while negatively with mean SpO_2_ (r = − 0.135, P = 0.035).

**Table 3:** Correlations between OCST parameters and MHR.

Variables	MHR	
	r	P value
AHI (events/h)	0.244*	< 0.001
Mean SpO_2_ (%)	− 0.135*	0.035
LSpO_2_ (%)	− 0.110	0.085
TS90 (%)	0.041	0.525
ODI	0.250*	< 0.001

*OCST* out of center sleep testing, *MHR* monocyte to high-density lipoprotein cholesterol ratio, *AHI* apnea–hypopnea index, *Mean* *SpO*_*2*_ mean oxygen saturation, *LSpO*_*2*_ lowest pulse oxygen saturation, *TS90* the percentage of sleep duration with SpO_2_ < 90%, *ODI* oxygen desaturation index.

*P value < 0.05.

Potential risk factors related to the presence and the severity of OSA were further investigated in both univariate and multivariate logistic regression analysis. Univariate logistic regression analysis showed that BMI (OR = 1.102, 95% confidence interval [CI]: 1.027–1.184, P = 0.007) and MHR (OR = 1.173, 95% CI 1.067–1.289, P = 0.001; Table 4) were associated with OSA; male sex (OR = 2.512, 95% CI 1.158–5.446, P = 0.020), BMI (OR = 1.118, 95% CI 1.046–1.194, P = 0.001), stage-2 hypertension (OR = 2.403, 95% CI 1.341–4.309, P = 0.003) and MHR (OR = 1.182, 95% CI 1.083–1.289, P < 0.001; Table 5) were associated with severe OSA. Further, multivariate logistic regression analysis identified that BMI and MHR were independently associated with the presence of OSA (BMI: OR = 1.081, 95% CI 1.005–1.161, P = 0.036; MHR: OR = 1.152, 95% CI 1.047–1.268, P = 0.009; Table 4), BMI, stage-2 hypertension and MHR were independently associated with severe OSA (BMI: OR = 1.089, 95% CI 1.016–1.168, P = 0.016; stage-2 hypertension: OR = 2.089, 95% CI 1.125–3.878, P = 0.020; MHR: OR = 1.142, 95% CI 1.041–1.252, P = 0.005; Table 5) after adjusted for other potential risk factors.

**Table 4:** Univariate and multivariate logistic regression analysis for the presence of OSA.

Variables	Univariate		Multivariate	
	OR (95% CI)	P value	OR (95% CI)	P value
Age	1.001 (0.979–1.023)	0.958		
Male	1.816 (0.982–3.359)	0.057		
BMI	1.102 (1.027–1.184)	0.007*	1.081 (1.005–1.161)	0.036*
Smoking	1.035 (0.588–1.824)	0.905		
Alcohol consumption	1.130 (0.580–2.203)	0.72		
Diabetes mellitus	0.726 (0.403–1.308)	0.286		
Stage-2 hypertension	1.509 (0.831–2.738)	0.176		
MHR	1.173 (1.067–1.289)	0.001*	1.152 (1.047–1.268)	0.009*

*BMI* body mass index, *MHR* monocyte to high-density lipoprotein cholesterol ratio.

*P value < 0.05.

**Table 5:** Univariate and multivariate logistic regression analysis for severe OSA.

Variables	Univariate		Multivariate	
	OR (95% CI)	P value	OR (95% CI)	P value
Age	0.982 (0.960–1.005)	0.122		
Male	2.512 (1.158–5.446)	0.020*	2.134 (0.949–4.797)	0.067
BMI	1.118 (1.046–1.194)	0.001*	1.089 (1.016–1.168)	0.016*
Alcohol consumption	1.241 (0.645–2.387)	0.518		
Smoking	1.453 (0.818–2.582)	0.202		
Diabetes mellitus	0.721 (0.382–1.361)	0.313		
Stage-2 hypertension	2.403 (1.341–4.309)	0.003*	2.089 (1.125–3.878)	0.020*
MHR	1.182 (1.083–1.289)	< 0.001*	1.142 (1.041–1.252)	0.005*

*BMI* body mass index, *MHR* monocyte to high-density lipoprotein cholesterol ratio.

*P value < 0.05.

**Figure 3:**
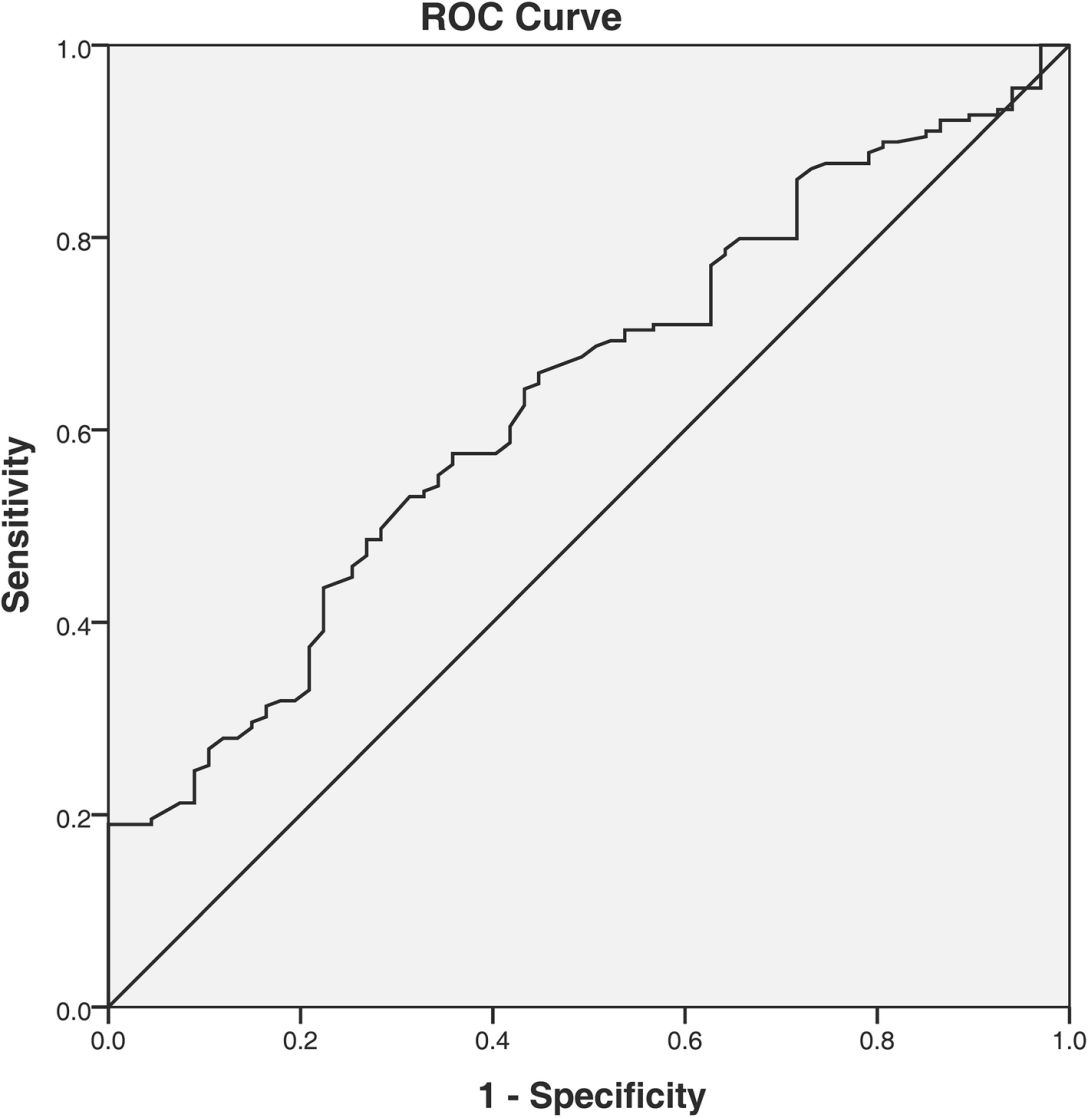
The ROC curve analysis for MHR in predicting the presence of OSA. The cut-off value of MHR was 10.3, with a sensitivity of 53.1% and a specificity of 68.7%, AUC: 0.634 (95% CI 0.560–0.708; P = 0.038). *ROC* receiver operating characteristic, *MHR* monocyte to high-density lipoprotein cholesterol ratio, *OSA* obstructive sleep apnea, *AUC* area under the curve.

**Figure 4:**
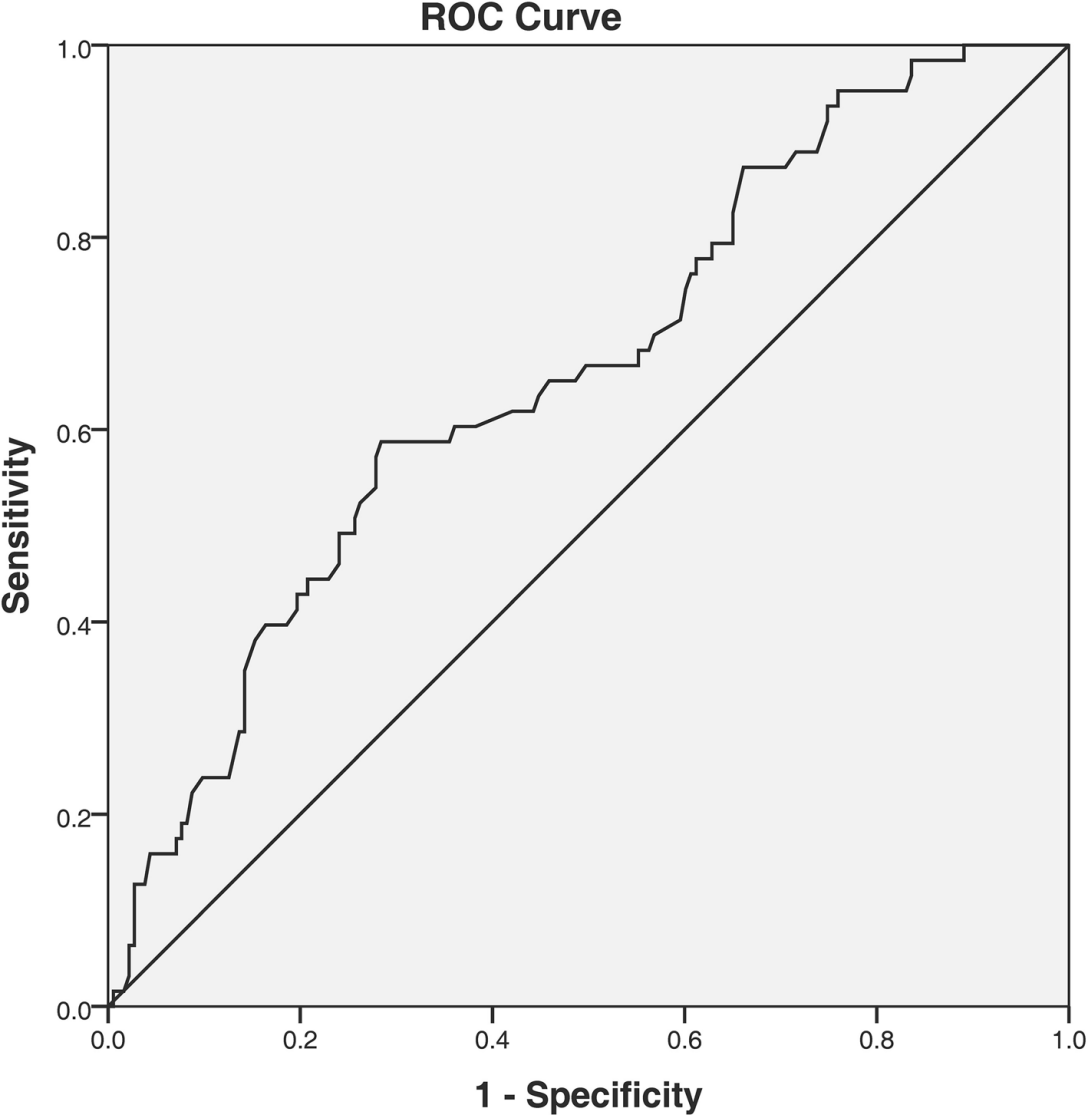
The ROC curve analysis for MHR in predicting severe OSA. The cut-off value of MHR was 11.4, with a sensitivity of 58.7% and a specificity of 71.6%, AUC: 0.660 (95% CI 0.583–0.737; P = 0.039). *ROC* receiver operating characteristic, *MHR* monocyte to high-density lipoprotein cholesterol ratio, *OSA* obstructive sleep apnea, *AUC* area under the curve.

For the prediction of OSA in hypertensive patients, the receiver-operating characteristic (ROC) curve analysis performed the cut-off value of MHR (> 10.3) with the greatest sum of sensitivity (53.1%) and specificity (68.7%), and area under the curve (AUC) of 0.634 (95% CI 0.560–0.708; P = 0.038; Fig. 3). In addition, the optimal MHR index cut-off value used for predicting severe OSA was 11.4 with the greatest sum of sensitivity (58.7%) and specificity (71.6%), and AUC of 0.660 (95% CI 0.583–0.737; P = 0.039; Fig. 4).

## Discussion

To our knowledge, this is the first article in the literature that evaluates the relationship between MHR and OSA in hypertensive patients. The results showed that MHR was significantly higher in hypertensive patients with OSA than those without OSA. MHR increased along with the severity of OSA. Moreover, we first demonstrated that MHR acted as an independent predictor of the presence and severity of OSA in hypertensive patients, suggesting that MHR could be used as a reliable parameter for OSA screening and severity evaluation in clinical practice of hypertension management.

Previous data showed that aging, male sex, BMI, smoking and alcohol consumption were strongly associated with OSA^11,12^. In our study of the hypertensive patients, there were no differences regarding aging, male sex, smoking and alcohol consumption between patients with and without OSA. However, BMI was found increasing with the severity of OSA, and was addressed as independent predictor of OSA, which was in agreement with results in the general population.

OSA has been described as a low-grade chronic inflammatory disease due to CIH, which could exacerbate the progression of hypertension^5^. Monocytes are essential immune cells that play a key role in the process of inflammation and oxidative stress. It has been proved that hypoxia could increase monocyte counts and the pro-inflammatory effects of monocytes. Alvarez-Martins et al*.* found that CIH increased monocyte counts via affecting hematopoiesis and the bone marrow microenvironment in a rat model of OSA^13^. Tamaki et al*.* found that the invasive ability of monocytes was significantly higher in patients with OSA compared with control subjects^14^. Additionally, our study in the hypertensive patients showed that monocyte counts were significantly higher in the severe OSA group^9,10^, indicating the obvious systemic inflammation of severe OSA.

Contrary to the inflammatory property of monocytes, accumulating evidence suggests the anti-inflammatory and antioxidative effects of HDL cholesterol via suppressing cytokines expression and inhibiting monocytes activation and extravagation^15,16^. Data from the European Sleep Apnea Database showed that HDL cholesterol was significantly reduced in the highest AHI quartile^17^. Our study of the hypertensive patients further found that the level of HDL cholesterol was the lowest in the severe OSA group, which might be attributed to the aggravated systemic inflammation induced by CIH.

In regard to the inflammatory property of monocytes and the anti-inflammatory property of HDL cholesterol, MHR was proposed as a new marker of systemic inflammation. A number of studies have implicated that MHR was independently associated with the occurrence and prognosis of several cardiovascular diseases. In patients with ST-segment elevation myocardial infarction (STEMI) treated with primary percutaneous coronary intervention (pPCI), MHR was reported as an independent predictor of stent thrombosis^18^, no reflow^19^, contrast-induced nephropathy^20^, and in-hospital mortality^21^. In the field of hypertension, MHR was also in accordance with asymptomatic organ damage and non-dipper hypertension^22,23^. Additionally, MHR was found independently predicted the late recurrence of paroxysmal AF after radiofrequency ablation, with the same predictive value as left atrial diameter^24^.

Limited studies have explored the relationship of MHR with OSA. Atan et al. found a dose–response correlation between MHR and the severity of OSA^8^. Both Li et al. and Inonu et al. reported the strong association of MHR with the occurrence of cardiovascular disease in OSA patients^9,10^. Those findings suggested that MHR was associated with OSA in general population. However, whether MHR is an independent indicator of the presence and the severity of OSA has not been demonstrated. Besides, none of the previous studies have ever investigated the association between MHR and OSA in patients with hypertension. Our study in hypertensive patients found that MHR levels correlated positively with AHI and ODI while negatively with mean SpO_2_. MHR values in OSA group was significantly higher than the control group. Moreover, MHR values in severe OSA group were the highest among all 4 groups. These findings were consistent with the results in general population^8–10^. By further logistic regression analysis, MHR was found independently associated with the presence of OSA and severe OSA in hypertensive patients in spite of sex, BMI and hypertension severity, indicating that elevated MHR value might be a predictor for the development and progression of OSA in hypertensive patients. Although the predictive significance of the optimal cut-off value of MHR for OSA was not strong due to the relatively small size of the study, the predictive power of MHR was moderately improved in the prediction for severe OSA, which might be attributed to the increased systemic inflammation in severe OSA patients.

Several limitations of our study should be mentioned. First, given the retrospective cross-sectional nature of this single center study, clinical factors not contained in this study might influence the results. Second, MHR changes before and after continuous positive airway pressure (CPAP) treatment were not investigated. Third, the sample size of this study was relatively small and OCST was used in OSA diagnosis and severity evaluation. Although OCST is widely used and reported comparable to full polysomnography (PSG) both in the diagnosis and severity evaluation of OSA in appropriate clinical settings, the absence of electroencephalogram may reduce the sensitivity of OCST which may result in the underestimation of sleep disordered breathing severity. The result conducting by OCST should be further validated in full PSG. Thus, further multi-center, prospective interventional clinical trials with CPAP treatment on larger populations are needed in the future.

In conclusion, our study found that in hypertensive patients, MHR increased with the severity of OSA, and independently associated with the presence and severity of OSA regardless of sex, BMI and hypertension severity. As OSA is an independent risk factor for hypertension and management of OSA could better control blood pressure and reduce consequent cardiovascular morbidities, MHR, a practical and cost-effective test, might be used as an available marker to evaluate OSA risk and severity in clinical management of hypertension.

## Methods

### Study population

We retrospectively analyzed consecutive patients who were diagnosed with hypertension and recorded the OCST based on the clinical suspicion of OSA at the cardiovascular department of Peking University Shougang Hospital from July 2016 to September 2019. The diagnosis of hypertension was made based on systolic/diastolic blood BP ≥ 140/90 mmHg, anti-hypertensive medication use, or a previous hypertension diagnosis. The study was in compliance with the principles outlined in the Declaration of Helsinki and approved by the local ethics committee of Peking University Shougang Hospital. Informed consent was obtained from all participants.

Patients younger than 18 years of age and with secondary hypertension other than OSAS, comorbid sleep disorders (e.g., central sleep apnea, restless leg syndrome, narcolepsy, insomnia, circadian rhythm disorders, etc.), neural-muscular disease, previous treatment for OSA (e.g., CPAP, surgery, and oral device, etc.), hypoxemic lung disease (e.g., chronic obstructive pulmonary disease, interstitial lung disease, asthma, etc.), hematologic disease, congestive heart failure, liver or kidney disease, malignancy, pregnancy, infection, autoimmune disease, and anti-inflammatory medication use were excluded as previous studies described^9,10,25^. In total, 246 subjects were included.

Demographic characteristics, systolic BP, diastolic BP, medication use, history of diabetes, dyslipidemia, CAD, smoking and alcohol consumption were retrospectively reviewed. BMI was calculated as the patient’s weight (kg)/height^2^ (m^2^). Hypertension severity was divided into 2 stages according to BP levels or medication use (stage-1: BP < 160/100 mmHg or BP under control with 1 or 2 antihypertensive drugs; stage-2: BP ≥ 160/100 mmHg or BP under control with ≥ 3 antihypertensive drugs)^26,27^.

### Laboratory measurements

Blood samples were obtained from the patients in the morning after 12 h of fasting. Monocyte was determined by flow cytometry with a Sysmex XN-2800 automated hematology analyzer (Japan). Lipid profiles including total cholesterol, triglycerides, HDL cholesterol and LDL cholesterol were analyzed with AU5811 automatic biochemical analyzer (Beckman Coulter, USA). MHR was calculated as the monocyte count (10^3^/µL)/HDL cholesterol (mg/dL).

### OCST evaluation

All participants performed OCST (Apnea Link Air, ResMed Germany Inc, Germany), which included the following: electrocardiography, pulse oxygen saturation, oral and nasal airflow, nasal air pressure, thoracic-abdominal respiratory movement, snoring microphone, and body position. The application of OCST in OSA screening and severity evaluation has been validated against full polysomnography^28,29^. Patients with comorbid conditions (including comorbid sleep disorders, neural-muscular disease, hypoxemic lung disease, congestive heart failure, etc.) that were not recommended to receive OCST instead of full PSG were excluded in this study^25^. OCST was programmed to record automatically, starting from 30 min after the patients went to bed. Recordings that last for less than 300 min were excluded. Polysomnography data were scored manually by trained personnel. According to American Association of Sleep Medicine (AASM) criteria, apnea was defined as a decrease to 0–20% of oronasal air flow for longer than 10 s; hypopnea was defined as a decrease of oronasal air flow by 50% for longer than 10 s, or a decrease of both oronasal air flow by at least 30% and oxygen saturation by 4% for longer than 10 s. The apnea and hypopnea counts per hour were recorded as the apnea–hypopnea index (AHI). The oxygen desaturation index (ODI) was defined as the number of oxygen level drops 3% from baseline per hour. Diagnosis of OSA was made solely when the AHI in the recorded study was ≥ 5 events per hour, irrespective of daytime OSA symptoms, which allowed objective evaluation of the disease severity^30^. According to the AHI, patients were categorized into the control group (AHI < 5) and the OSA group (AHI ≥ 5). Then the OSA group was further categorized into the mild (AHI: 5–14.9), moderate (AHI: 15–29.9), and severe (AHI ≥ 30) OSA group. The percentage of sleep duration with SpO_2_ < 90% (TS90), lowest pulse oxygen saturation (LSpO_2_), mean oxygen saturation (mean SpO_2_), and ODI were also included.

### Statistics

The results were expressed as mean ± SD, median (interquartile range), or number (percentage). Continuous variables were investigated for normal distribution with histograms, probability plots and Kolmogorov–Smirnov test. Differences between groups was assessed by Student unpaired t-test and one-way analysis of variance for normally distributed data; and Mann–Whitney U test and Kruskall–Wallis H test for non-normally distributed data, respectively. Comparison of categorical variables was analyzed by chi square test. Correlations were assessed by Pearson’s rank correlation. The effect of various variables on OSA risk and OSA severity was analyzed with univariate and multivariate logistic regression analysis. Receiver-operating characteristic (ROC) curve with Youden index was used to estimate the predictive validity and determine the optimal MHR cut-off value. P value < 0.05 was considered statistically significant. Statistical analysis was carried out using SPSS version 22.0 (IBM SPSS Statistics for Windows, USA).

## Data Availability

The dataset generated and analyzed in this study are available from the corresponding author upon reasonable request.
